# OXPHOS-dependent metabolic reprogramming prompts metastatic potential of breast cancer cells under osteogenic differentiation

**DOI:** 10.1038/s41416-020-01040-y

**Published:** 2020-09-16

**Authors:** Yangling Hu, Weimin Xu, Hui Zeng, Zilong He, Xiao Lu, Daming Zuo, Genggeng Qin, Weiguo Chen

**Affiliations:** 1 Department of Radiology, Nanfang Hospital, Southern Medical University, 510515 Guangzhou, China; 2 Department of Immunology, School of Basic Medical Sciences, Southern Medical University, 510515 Guangzhou, China; 3 Institute of Molecular Immunology, School of Laboratory Medicine and Biotechnology, Southern Medical University, 510515 Guangzhou, China

**Keywords:** Cancer metabolism, Breast cancer, Metastasis

## Abstract

**Background:**

Microcalcification is one of the most reliable clinical features of the malignancy risk of breast cancer, and it is associated with enhanced tumour aggressiveness and poor prognosis. However, its underlying molecular mechanism remains unclear.

**Methods:**

Clinical data were retrieved to analyse the association between calcification and bone metastasis in patients with breast cancer. Using multiple human breast cancer cell lines, the osteogenic cocktail model was established in vitro to demonstrate calcification-exacerbated metastasis. Migration and invasion characteristics were determined by wound healing and transwell migration. mRNA and protein expression were identified by quantitative PCR and western blotting. Metabolic alterations in breast cancer cells were evaluated using Seahorse Analyser.

**Results:**

The osteogenic differentiation of human breast cancer cells activated the classical TGF-β/Smad signalling pathway and the non-canonical MAPK pathway, which, in turn, exacerbated the progression of epithelial–mesenchymal transition (EMT). The metabolic programme switched to enhancing mitochondrial oxidative phosphorylation (OXPHOS) upon osteogenic differentiation. Rotenone was used to inhibit the OXPHOS complex during osteogenesis to block mitochondrial function, consequently reversing the EMT phenotype.

**Conclusions:**

This study provides important insights into the mechanisms involved in breast cancer bone metastasis, and outlines a possible strategy to intervene in OXPHOS for the treatment of breast tumours.

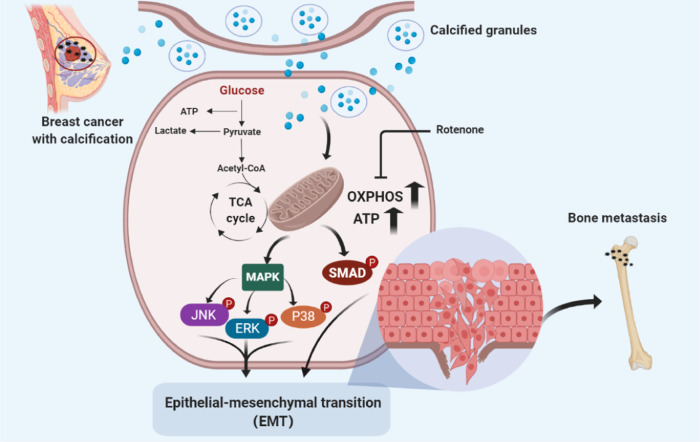

## Abstract (web summary)

This manuscript provides a simplified overview of the involvement of mitochondrial activity and OXPHOS in the metastasis of breast cancer with calcification. We propose that massive accumulation of calcified granules due to breast cancer necrosis or deposition of spontaneous secretions around the glands lead to reprogramming of tumour metabolism, particularly enhancement of mitochondrial metabolism. Upregulation of the OXPHOS complex and subsequent activation of TGFβ-SMAD and MAPK (e.g., JNK, ERK, P38) signalling pathways facilitate the EMT process of breast cancer cells. Interestingly, the OXPHOS inhibitor rotenone could block the EMT process due to cell calcification.

## Background

Breast cancer is the most frequently diagnosed cancer and the leading cause of cancer-related death among women worldwide.^1,2^ Breast tumours can be detected via mammograms; however, a common feature is the presence of small deposits of calcium called microcalcification, which may be the only radiographically detectable sign of breast neoplasm in many cases.^3,4^ Several non-palpable breast tumours and up to 90% of ductal carcinomas in situ (DCIS) present with radiographically dense calcification in mammographic scans.^5^ Casting-type calcification is an independent prognostic factor for breast cancer. Two species of mammary calcifications have been identified, namely calcium oxalate and hydroxyapatite (HA), at the site of breast cancer. Calcium oxalate is mostly associated with benign lesions, whereas HA is related to both benign and malignant breast tumours.^6^ Calcifications represent one of the most reliable clinical signs of malignancy in breast cancer, and previous studies have revealed that HA potentially enhances breast tumour progression and malignancy. Therefore, the determination of the molecular mechanisms of calcification formation and evaluation of its clinical significance in the development of breast cancer are important.

The skeleton is the most common destination for breast cancer migration and colonisation.^7,8^ Previous studies have shown that bone metastasis affected >70% of patients with advanced breast cancer,^9^ and that breast cancer with calcification is more prone to bone metastasis.^10–12^ Moreover, the association between calcification-related molecules (e.g., BMP2, RUNX2, OPN, BSP and ALP) and the development and metastasis of breast cancer, particularly in disease-specific bone metastasis, has been reported.^13–19^ Such calcification-related genes that may lead to metastasis and poor prognosis cannot be ignored. However, the precise mechanism of the relationship between calcification and metastasis is still unclear. Metabolic reprogramming is a hallmark of cancer and cell transformation. Changes in cellular metabolism were thought to be the consequence of cancer rather than playing a major role in its development and promotion.^20^ Recently, it has been found that cancer metabolic reprogramming is critical for the growth and maintenance of cancer cells. Mitochondria are dynamic intracellular organelles that maintain the biosynthetic as well as energetic capacities of cancer cells, and play a critical role in cellular metabolism by producing ATP through the oxidative phosphorylation system (OXPHOS).^21–23^ Breast cancer cells exhibit extensive metabolic heterogeneity. Normal and breast cancer-invasive ductal carcinoma cells show differential expression of genes responsible for mitochondrial metabolism, and the metabolic process is associated with their metastatic sites.^21^ A recent study has established that the triple-negative breast cancer cell line mainly exhibits enhanced OXPHOS, which is important for its bone metastasis.^24^ Whether metabolic alterations in breast cancer with calcification affect subsequent metastasis or prognosis remains unclear.^25–28^ The mechanism of metastasis in breast cancer with calcification and its relationship with metabolism warrant investigation.

In this study, we sought to investigate the different intrinsic metabolic profiles and the capacity of breast cancer cells to undergo epithelial-to-mesenchymal transition (EMT) during calcification. We observed that OXPHOS plays a critical role as a potential intermediate bridge between calcification and EMT, suggesting that interfering with OXPHOS helps limit breast cancer metastasis.

## Methods

### Patient characteristics

A total of 404 women admitted to the Department of Breast Surgery who were pathologically confirmed to have breast cancer in Nanfang Hospital, Southern Medical University, were recruited. All patients had undergone whole-body bone scan in the Department of Nuclear Medicine from June 2009 to September 2015. Clinicopathological information was obtained from the patients’ medical records, including age, menopausal status, pathological type, lymph-node status, histological grade and expressions of oestrogen receptor (ER), progesterone receptor (PR) and epidermal growth factor receptor 2 (HER2). The patients were divided into two groups based on the presence or absence of calcifications on mammography. None of the patients had confirmed metastasis or received radiotherapy or chemotherapy before surgery. Diagnosis of bone metastasis was determined from the medical records of the inpatients and outpatients, or by consulting the general practitioners of the patients. The results of whole-body bone scan were classified as negative or positive for bone metastasis. Negative bone metastasis was defined as being clinically free of bone metastasis for more than 2 years. For patients with ambiguous findings and definite abnormal uptake on whole-body bone scans, other imaging modalities, including plain X-ray, computed tomography and magnetic resonance imaging, were used to confirm the diagnosis of bone metastasis. Patients with other malignant neoplasms, humoral hypercalcaemia, hyperthyroidism, hyperparathyroidism, hypoparathyroidism, renal failure, traumatic fracture (within 6 months), a history of gastrectomy or postmenopausal use of hormone-related humoral hypercalcaemia were excluded. All patients provided informed consent before enrolment, and the study was approved by the ethics committee of Nanfang Hospital, Southern Medical University (reference number: NFEC-201706-K3-01).

### Cell culture

Human breast carcinoma cell lines MDA-MB-468, MCF-7 and MDA-MB-231 were purchased from Cellcook Biotech Co., Ltd (Guangzhou, China) and cultured in DMEM (Gibco, Thermo Scientific, USA) supplemented with 10% foetal bovine serum (FBS) and 1% penicillin–streptomycin at 37 °C in a humidified incubator with 5% CO_2_.

### Osteogenic cocktail (OC) and in vitro cell calcification

Cells were seeded into six-well culture plates (day 0) at a density of (1–2) × 10^5^ cells per well. The next day (day 1), the cells were treated with culture or calcification medium containing 50 µg/mL ascorbic acid (Sigma-Aldrich, #A4403), 10 mM β glycerine sodium phosphate (Sigma-Aldrich, G6501) and 10–100 nM dexamethasone (Sigma-Aldrich, #D4902). Cells were cultured for 3, 5, 7 and 14 days, and the media were replaced every 2–3 days for detection of calcification indicators. For detection of other indicators, cells were plated at the same time and harvested uniformly on day 7, and appropriate stimulation time points were arranged according to the duration of induction. Induction with OC for 0 day was considered as 7 days of culture control. If the cells were subjected to induction with OC for 3 days, the OC medium was changed on day 4, and the cells were harvested on day 7.

### Alizarin red S staining and quantification of calcification

Calcification was assessed on days 3, 5, 7 and 14. The culture or calcification medium on the 3-cm dish was removed; the cells were washed with PBS thrice and fixed with 4% paraformaldehyde for 30 min at room temperature. The fixed cells were washed thrice for 5 min, stained with alizarin red S (2%, pH 4.4) for 5 min and washed thrice for 5 min in PBS. Five randomly selected fields per well were imaged on the microscope. Finally, the cells were incubated at room temperature in 100 mM cetylpyridinium chloride for 1 h. Dissolved calcium media were read at an absorbance of 562 nm.

### ALP-activity detection

After 3, 5, 7 and 14 days, MDA-MB-468 and MCF-7 cells were washed with PBS and lysed using RIPA lysis buffer (Beyotime Institute of Biotechnology, Beijing, China). Alkaline Phosphatase Assay Kit (Beyotime) was used to evaluate the activity of alkaline phosphatase, according to the manufacturer’s instructions.

### Total RNA extraction, cDNA conversion and quantitative real-time (qRT)- PCR

The total RNA was extracted from the human breast cancer MDA-MB-468 and MCF-7 cells at different conditions using Trizol Reagent (TransGene Biotech, Beijing, China). qRT-PCR was performed on a 7900HT Fast Real-Time PCR System (Applied Biosystems, San Francisco, CA, USA) using SYBR Green assays (TransGene Biotech), according to the manufacturer’s instructions. The relative mRNA expression of transcripts was normalised to β-actin and analysed using the 2^−∆∆Ct^ method. Primer sequences are listed in Supplementary Table 3.

### Western blotting

After treatment, MDA-MB-468 and MCF-7 cells were harvested, and protein extracts were prepared on ice with cold RIPA buffer containing protease inhibitor cocktail (Beyotime Biotechnology). Protein concentration was quantified, and protein was separated by 10% sodium dodecyl sulfate-polyacrylamide gel electrophoresis and then blotted onto polyvinylidene fluoride membranes (Beyotime). After blocking with 5% BSA in 1× Tris-buffered saline with 0.05% Tween-20 for 1.5 h at room temperature, the membranes were incubated with primary antibodies overnight at 4 °C. The next day, the membranes were incubated with appropriate dilution of the HRP-conjugated secondary antibody for 1 h at room temperature. SuperSignal West Femto Substrate (ECL detection kit, Thermo Scientific, Norwalk, CT, USA) was used to detect the protein expression. Images were captured on a film processor. All western blot experiments were repeated multiple times, with β-actin being used as a protein-loading control. Antibodies against the following proteins were obtained, as indicated: E-cadherin, N-cadherin, vimentin, α-SMA, PGC-1α and β-actin (Proteintech, China); RUNX2 and OPN (Santa); BSP, BMP2, OXPHOS, P-Smad2/3 and Smad2/3 (Abcam); HK I, HK II, PFKP, LDHA, PDHA, Snail, Twist, P-JNK, JNK, P-ERK, ERK, P-P38 and P38 (Cell Signaling Technology®).

### ELISA

To detect the secretion of transforming growth factor-β1 (TGF-β1), the cell supernatants were collected and analysed using a TGF-β1 ELISA kit (R&D Systems), following the manufacturer’s instructions.

### Scratching

For wound-healing assay, 5 × 10^5^ MDA-MB-468 and MCF-7 cells (day 0) were seeded on the six-well plates. When cell confluency reached ~90%, wounds were created in the confluent cells using a sterile 10-µL pipette tip. The cells were rinsed with PBS to remove any free-floating cells and debris. The cells were pre-treated with 10 µg/mL mitomycin C for 2 h and washed five times with PBS. A different medium was subsequently added, and the culture plates were incubated at 37 °C. Wound healing within the scrape lines was observed and photographed under the microscope at 0 and 4 days.

### Transwell

For migration assay, 2 × 10^5^ MDA-MB-468 cells (day 5) and MCF-7 cells (day 4) were added into the top chamber of the transwell plates (Corning Life Sciences, Corning, NY, USA) in 200 μL of DMEM without FBS and allowed to migrate for 36 h. The lower chamber was loaded with 600 µL of DMEM or conditioned medium containing 10% FBS as a chemoattractant. After incubation for 36 h, transmigrated cells (on the lower surface of the membrane) were fixed with methanol for 30 min, stained with 0.1% crystal violet for 20 min at room temperature, photographed and counted under a microscope in five random fields. For invasion assay, 2 × 10^5^ MDA-MB-468 and MCF-7 cells were seeded into the upper transwell chamber coated with diluted Matrigel (Corning) and allowed to invade for 48 h. The remaining experimental procedures were performed after the cell migration assay. Five randomly selected fields per filter were counted.

### Metabolic assays

The real-time mitochondrial oxygen consumption rate (OCR) and extracellular acidification rate (ECAR) were measured using the XF96 Extracellular Flux Analyser (Seahorse Bioscience) with the Cell Mito Stress Kit (Seahorse), as per the manufacturer’s instructions. MDA-MB-468 cells in osteogenic differentiation for 5 days and undifferentiated control cells were used for metabolic analysis. About (8–10) × 10^3^ cells per well were seeded in XFe96 cell culture plates and incubated for 24 h in F medium at 37 °C in a humidified atmosphere with 5% CO_2_. Before the assay, cells were rinsed twice and placed in a prewarmed XF assay medium (pH 7.4) at 37 °C in a non-CO_2_ incubator for 1 h. Subsequently, for measurement of ECAR, 10 mM glucose, 1 µM oligomycin and 50 mM 2-DG in XF assay medium were loaded into the injection ports in the XFe96 sensor cartridge to determine the ECARs. For measurement of mitochondrial oxygen consumption, the respiratory rate was measured at 37 °C by using the following perturbation drugs in succession: 1 µM oligomycin, 2 µM carbonyl cyanide-p-trifluoromethoxyphenylhydrazone and 0.5 µM rotenone/antimycin A. The basal OCR was measured before drug exposure. The experiment was performed with at least three replicates per condition.

### Lactate dehydrogenase (LDH) release assay

Release of LDH by the cells was confirmed using an LDH assay kit (Beyotime).

### Measurement of reactive oxygen species (ROS)

Cells (1 × 10^5^ cells/well) from each experimental condition were stained with DCFH-DA (10 µM) at 37 °C for 30 min. Intracellular ROS production was evaluated by flow cytometry (BD Biosciences, San Jose, CA, USA). Values were expressed by average fluorescence intensity, and data were analysed using FlowJo software (TreeStar, Ashland, OR, USA). Mitochondrial ROS were detected under the microscope. Cells were washed twice with PBS and labelled at 37 °C for 30 min with fluorescent probes (10 µM JC-1, a sensitive marker of mitochondrial membrane potential).

### Cell-proliferation assay

Breast cancer cells MDA-MB-468 and MCF-7 were seeded into 96-well culture plates at a density of 1 × 10^4^ cells per well. Cells were then treated with the OC medium and incubated at 37 °C and 5% CO_2_ for 4 days. After the addition of 10 µL of Cell Counting Kit-8 (CCK-8, ApexBio, Shanghai, China) solution to each well, the cells were incubated for an additional 1–2 h, according to the manufacturer’s instructions. The absorbance value was read using a microplate reader (Bio-Rad, Hercules, CA, USA) at 450 nm, and the data were plotted for the cell-proliferation curve. The experiment was repeated at least thrice.

### Statistical analysis

Chi-squared test was used to evaluate the association between calcification features and clinicopathological parameters. *P* < 0.05 was considered statistically significant. Through binary logistic regression, univariate and multivariate analysis were used to determine the risk factors that independently contributed to bone metastasis in patients with breast cancer. The experimental data were presented as the mean ± SD from at least three independent experiments. *P* values were calculated using two-tailed unpaired Student’s *t* test or one-way ANOVA with Bonferroni post-test correction. *P* < 0.05 was considered to indicate a statistically significant difference (*P < 0.05, **P < 0.01, ***P < 0.001). Statistical analyses were performed using SPSS 20.0 statistical package software (SPSS, Chicago, IL, USA) or GraphPad Prism 7.0 software (GraphPad Software Inc., San Diego, CA, USA).

### Conclusions

Our study describes the molecular mechanism that underlies the association of the formation of calcification in breast cancer with its metastatic capacity. EMT was strongly induced in breast cancer cells upon osteogenic differentiation. Calcified breast cancer cells showed elevated OXPHOS activity. OXPHOS might be a key link for calcification and cell metastasis, as OXPHOS significantly inhibited the EMT phenotype of breast cancer cells upon osteogenic differentiation. Overall, targeting OXPHOS appears a promising approach for limiting breast cancer metastasis.

## Results

### Relationship between calcification and clinicopathological features of breast cancer

Firstly, the association between mammographic calcification and clinicopathologic factors in patients with breast cancer was analysed (Supplementary Table 1). Breast cancer with calcification was more malignant, and axillary lymph-node involvement was more common in breast cancers with calcification than in those without calcification (56.8% vs. 40.4%, *P *= 0.001). In addition, tumours with calcification were more prone than tumours without calcification to bone and other distant metastases (17.5% vs. 7.1%, *P* = 0.001; 20.9% vs. 9.6%, *P* = 0.002). High tumour-proliferation index Ki-67 was also more common in breast cancers with calcification (80.7% vs. 71.3%, *P* = 0.039). There was a borderline difference of menopausal status between the group of patients with calcified and non-calcified tumours (42.8% vs. 33%, *P* = 0.046). Univariate analysis showed that calcification was greatly associated with bone metastasis in patients with breast cancer (OR = 2.783, *P* = 0.002). Multivariate logistic regression analysis demonstrated that axillary lymph-node status (OR = 4.329, *P* < 0.001) and calcification (OR = 2.522, *P* = 0.013) were independent risk factors for development of bone metastasis in patients with breast cancer (Supplementary Table 2). Age, menstrual status, Ki-67, ER status, HER2 status, PR status, grade, gland density and pathological type showed no significant relationship with bone metastasis. The odds ratio for these factors is listed in Supplementary Table 2.

**Fig. 1:**
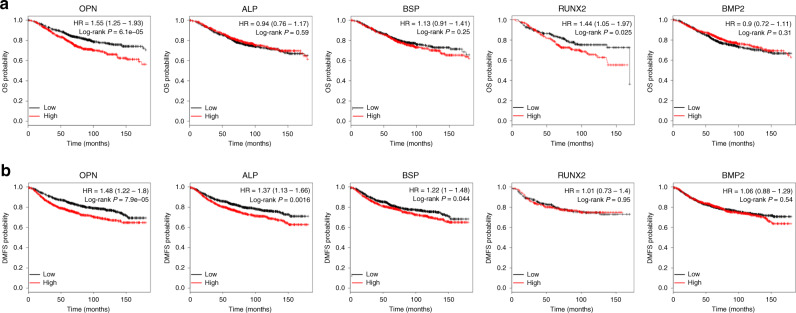
Calcification is associated with adverse clinical prognostic factors in patients with breast cancer. Breast cancer samples were stratified as high and low according to the expression of calcification-related genes, with median gene expression as the cut-off. Prognosis data were acquired and analysed using an online tool (http://www.kmplot.com). The overall survival (OS) rate (**a**) and distant metastasis-free survival (DMFS) rate (**b**) between the groups were compared using the log-rank test.

Next, we analysed the relationship of calcification-related genes (e.g., OPN, ALP, RUNX2, BMP2 and BSP) with overall survival (OS) and tumour metastasis using an online tool (http://www.kmplot.com). Overexpression of OPN and RUNX2 was found to be negatively correlated with the OS of patients with breast cancer (Fig. 1a). Moreover, the association of calcification-related genes with distant metastasis-free survival (DMFS) in patients with breast cancer was evaluated. Compared with low expression of OPN and ALP, high expression of the genes was associated with a significantly shorter DMFS in patients with breast cancer (Fig. 1b). Overexpression of BSP was mildly related to a poor DMFS (Fig. 1b). Altogether, the result gives us a hint that high expression of certain calcification-related genes may be related to a poor prognostic outcome in breast cancer.

### Human breast carcinoma cells form calcifications in vitro

**Fig. 2:**
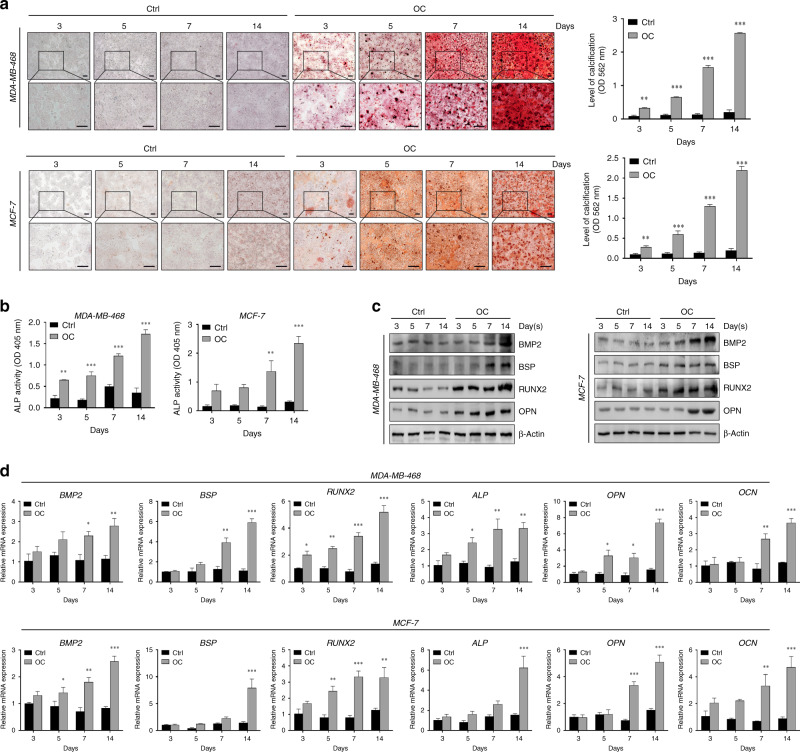
Detection of calcification in vitro. **a** Calcification of breast cancer cells was measured with alizarin red S staining at different time points after OC administration. Scale bars = 200 µm. **b** The ALP activity was measured at different time points after addition of OC in MDA-MB-468 and MCF-7 cells. **c** Western blotting of the calcification-related genes at the indicated time upon induction with OC. **d** mRNA levels of the corresponding molecules in MDA-MB-468 and MCF-7 cells were detected using quantitative RT-PCR and expressed as a ratio to β-actin. *P < 0.05, **P < 0.01, ***P < 0.001, NS not significant, determined by unpaired Student’s *t* test and one-way ANOVA with Bonferroni post-test correction. Data are shown as mean ± SD, and are representative of three independent experiments with similar results.

To investigate the molecular mechanisms of breast cancer calcification, we established a model for simulating the microcalcification of breast cancer cells in vitro. Human breast carcinoma cells MDA-MB-468 (basal-like), MDA-MB-231 (triple-negative) and MCF-7 (luminal type) were grown in media supplemented with an osteogenic cocktail (OC, consisting of β-glycerophosphate, ascorbic acid and dexamethasone, respectively). Positive staining was detected in the OC groups using alizarin red S. Further, alizarin red S was extracted with 10% cetylpyridinium chloride, and the concentration of dye in the extract was measured at an optical density (OD) of 562 nm. The intensity of staining increased over time, with the strongest staining observed in the OC group (Fig. 2a). Interestingly, MDA-MB-468 cells are similar to MCF-7 cells, both of which are more sensitive than MDA-MB-231 cells to osteogenic differentiation (Fig. 2a and Supplementary Fig. 1a). In addition, a statistically significant increase in ALP activity, a key indicator of calcification, was detected in the OC-treated MDA-MB-468 and MCF-7 cells (Fig. 2b). Immunoblotting analysis showed that calcification-related molecules were markedly elevated in both OC-treated cells (Fig. 2c). Further, quantitative RT-PCR (qRT-PCR) confirmed that the calcification-associated biomarkers were increased in the MDA-MB-468 and MCF-7 cells cultured with calcification medium (Fig. 2d). Collectively, these data suggest that OC promotes the calcification of breast cancer cells in vitro.

### Enhanced metastatic capacity and activated EMT-related signalling pathways in breast cancer cells under osteogenic induction

**Fig. 3:**
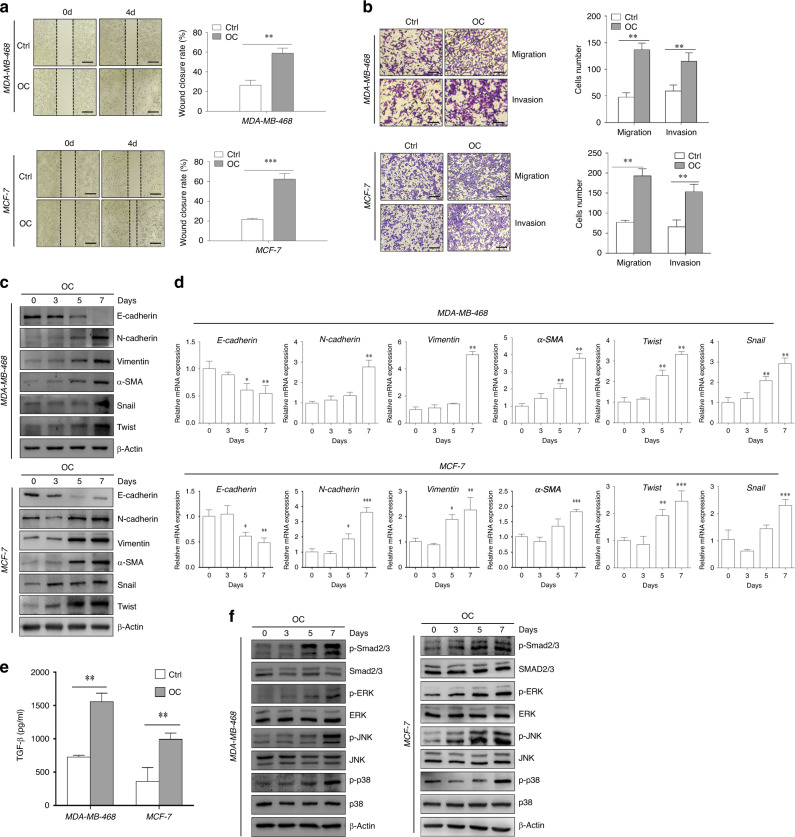
Enhancement of metastatic ability and activation of epithelial-to-mesenchymal transition (EMT)-related signalling pathways in breast cancer cells after osteogenic induction. **a** Cell migration was measured by scratch-wound analysis at the indicated time. Scale bars = 500 µm. **b** The migration and invasion capacities were determined by transwell assay and quantified by counting the number of cells perforated crystal violet at the indicated time points after OC treatment. Scale bar = 200 µm. Western blotting (**c**) and qRT-PCR (**d**) of EMT-related genes in MDA-MB-468 and MCF-7 cells at various time points after OC induction. **e** Transforming growth factor (TGF)-β levels in MDA-MB-468 and MCF-7 cell supernatants were assessed by collecting and mixing supernatants at days 3 and 5 of OC induction. **f** The activation of the EMT-related Smad2/3 and MAPK (e.g., JNK, ERK and p38) pathway in MDA-MB-468 and MCF-7 cells was measured by western blotting at various time points upon OC treatment. β-actin was used as an internal control. *P < 0.05, **P < 0.01, determined by unpaired Student’s *t* test and one-way ANOVA with Bonferroni post-test correction. Data are shown as mean ± SD, and they represent three independent experiments with similar results.

The metastatic ability of breast cancer cells with calcification was further explored by scratch, migration and transwell assays. The morphology of MDA-MB-468 and MCF-7 cells grown in the OC medium gradually changed from round or small squares to long spindles (Supplementary Fig. 2). Compared with the corresponding control cells, the migration of MDA-MB-468 and MCF-7 cells markedly increased under the calcification conditions (Fig. 3a). In addition, the invasion ability of breast cancer cells was enhanced by treatment with the calcification medium (Fig. 3b). It is well known that EMT enables cancer cells to acquire the abilities of invasion and metastasis. Compared with control cells, OC-treated breast cancer cells showed reduced levels of epithelial markers (E-cadherin) and increased levels of mesenchymal markers (vimentin, N-cadherin, α-SMA, Snail and Twist) (Fig. 3c, d).

The TGF-β signalling pathway plays an important role in regulation of EMT and activation of metastatic cancer cells. Increased TGF-β secretion was observed in the OC-stimulated breast cancer cells (Fig. 3e). In addition, the phosphorylation of Smad2/3 was significantly increased in OC-treated MDA-MB-468 and MCF-7 cells, whereas the total protein levels of Smad2/3 did not change (Fig. 3f). TGF-β also activates the MAPK signalling pathway during the EMT process.^29^ We investigated the signalling pathways and found increased phosphorylation of MAPKs (e.g., JNK, ERK and p38) in OC-treated MDA-MB-468 and MCF-7 cells (Fig. 3f). CCK-8 assay indicated that the OC had little effect on cell proliferation (Supplementary Fig. 3). Collectively, these results indicate that breast cancer cells exhibit an increased capacity of tumour migration and invasion during osteogenic differentiation.

### Minor alteration in glycolysis metabolic activity during osteogenic differentiation

**Fig. 4:**
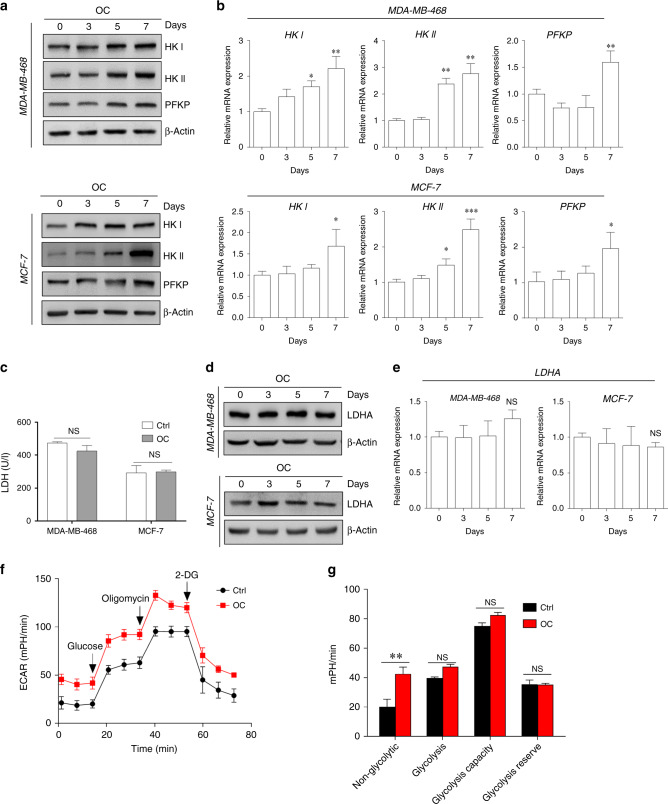
Minor alterations in glycolysis metabolism of breast cancer cells during osteogenic differentiation. Western blotting (**a**) and quantitative real-time (qRT)-PCR (**b**) were conducted to evaluate the expressions of selective glycolysis-related genes (e.g., HK I, HK II and PFKP) in MDA-MB-468 and MCF-7 cells at different time points after OC treatment. **c** Lactate dehydrogenase (LDH) activity in the cells was measured during 4 days of OC treatment. The expression of LDHA in the cells was assessed by western blotting (**d**) and qRT-PCR (**e**). **f** Seahorse analysis of extracellular acidification rate (ECAR) in MDA-MB-468 cells at specific time points induced by OC. **g** ECAR of non-glycolysis, glycolysis, glycolytic capacity and the glycolytic reserve was analysed. *P < 0.05, **P < 0.01, NS not significant, determined by unpaired Student’s *t* test and one-way ANOVA with Bonferroni post-test correction. Data are shown as mean ± SD, and they represent three independent experiments with similar results.

Alterations in cell metabolism have been associated with tissue calcification.^30^ We evaluated the glucose metabolism of cancer cells under osteogenic differentiation. Western blotting revealed increased levels of genes involved in glucose metabolism in breast cancer cells cultured with OC, compared with those cultured without OC (Fig. 4a). In addition, the alteration of glucose metabolism-related genes in MDA-MB-468 cells subjected and not subjected to OC treatment was similar to that observed in MCF-7 cells (Fig. 4b).

However, the activity of LDH (Fig. 4c) and expression of lactate dehydrogenase A (LDHA) (Fig. 4d, e) were comparable between OC-treated breast cancer and control cells. Compared with control cells, OC-treated cells exhibited slightly increased ECARs, determined using Seahorse XFe96 Extracellular Flux Analyser (Seahorse Bioscience, Billerica, MA, USA) (Fig. 4f). However, there was no statistically significant difference in the quantitative analysis of glycolysis factors between the OC-treated and control groups (Fig. 4g). These findings indicate that alteration in the metastatic properties of cancer cells during calcification may not depend on glycolysis.

### Increased activity of mitochondrial metabolism in breast cancer cells under osteogenic induction

**Fig. 5:**
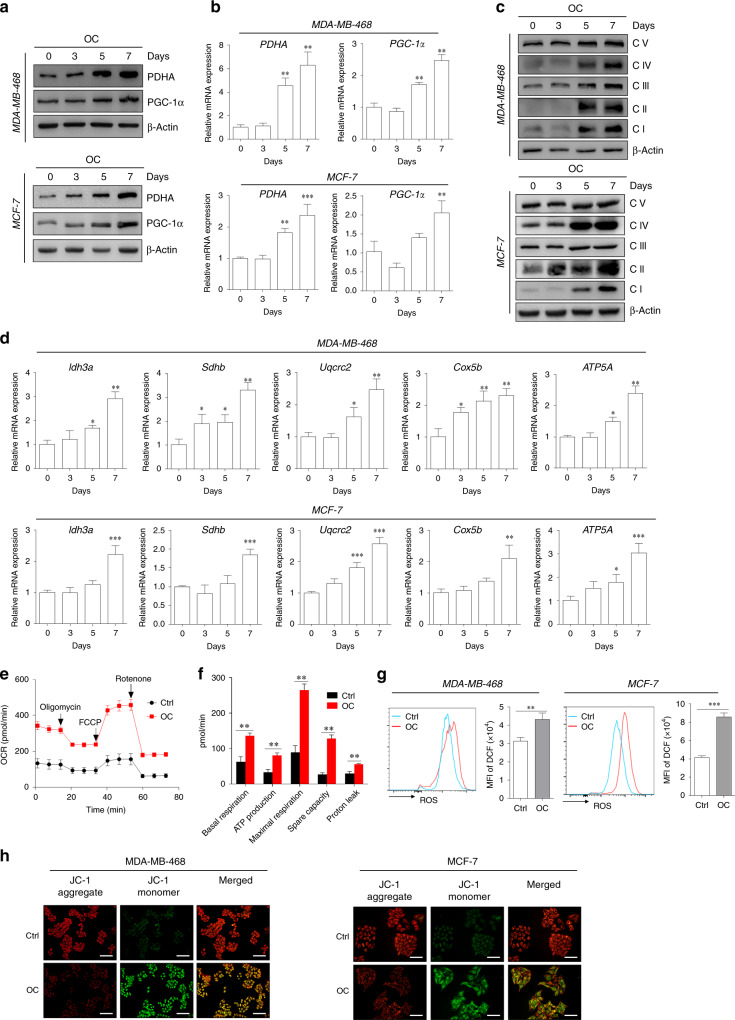
Enhancement of mitochondrial metabolism in the calcification-mediated epithelial-to-mesenchymal transition (EMT) model. The expressions of mitochondrial genes (e.g., PDHA and PGC-1α) of MDA-MB-468 and MCF-7 cells at different induction time points were evaluated by western blotting (**a**) and quantitative real-time (qRT)-PCR (**b**). The levels of OXPHOS complex subunits (**c**) were evaluated using western blotting at the corresponding induction time. **d** mRNA levels of Idh3a, Sdhb, Uqcrc2, Cox5b and ATP5A representing the OXPHOS complex were determined using qRT-PCR. β-actin was used as an internal control. **e** Seahorse analysis of oxygen consumption rate (OCR) in MDA-MB-468 cells at specific time points induced by OC. **f** OCR of basal and maximal mitochondrial respiration, ATP production, spare capacity and proton leak was analysed. **g** Flow-cytometry analysis of the intercellular reactive oxygen species (ROS) level by the fluorescent probe DCFH-DA in 7 days following OC treatment. **h** Alterations of mitochondrial membrane potential in breast cancer cells were measured by membrane-permeant JC-1 dye staining. Scale bars = 100 µm. *P < 0.05, **P < 0.01, ***P < 0.001; NS not significant, determined by unpaired Student’s *t* test and one-way ANOVA with Bonferroni post-test correction. Data are demonstrated as mean ± SD, and they represent three independent experiments with similar results.

The altered metabolic phenotype has been recognised as a hallmark of tumour cells for many years, and the reprogramming of energy metabolism contributes to aggressive cancer phenotypes. PDHA is the rate-limiting step in the conversion of pyruvate to acetyl-CoA, which enters the tricarboxylic acid (TCA) cycle and increases oxygen consumption and ATP production in the mitochondria. Compared with control cells, OC-treated cells exhibited greatly increased expression of PDHA at both protein and mRNA levels (Fig. 5a, b). Similarly, PGC-1α, a central inducer of mitochondrial biogenesis, was upregulated in the OC-treated breast cancer cells (Fig. 5a, b). We further observed that OC treatment substantially enhanced the expression of respiratory complexes I–V (i.e., NADH dehydrogenase, succinate dehydrogenase, ubiquinol dehydrogenase, cytochrome c oxidase and ATP synthase or FoF1-ATPase) in MDA-MB-468 and MCF-7 cells (Fig. 5c). OC stimulation also increased the expressions of PDHA, PGC-1α and respiratory complexes in MDA-MB-231 cells (Supplementary Fig. 1b, c). In addition, the mRNA expressions of OXPHOS-related genes (e.g., Idh3a, Sdhb, Cox5b, Uqcrc2 and ATP5A) were greatly increased in OC-treated cells, compared with control cells (Fig. 5d). Next, we evaluated whether OC administration influences mitochondrial respiration in MDA-MB-468 cells. We found that OC-treated cells exhibited significantly increased basal and maximum mitochondrial respiration (Fig. 5e, f). The ATP-producing capacity of OC-cultured cells was also increased, compared with that of control cells (Fig. 5f). Compared with control cells, treatment with OC resulted in markedly increased mitochondrial activity, as indicated by increased levels of ROS (Fig. 5g), and limited mitochondrial membrane potential (Fig. 5h). The results suggest that OC treatment increased metabolic capacity, especially mitochondrial metabolism activity, in breast cancer cells.

### Blockade of OXPHOS activity reversed aggressive cancer phenotypes of breast cancer cells during osteogenic differentiation

**Fig. 6:**
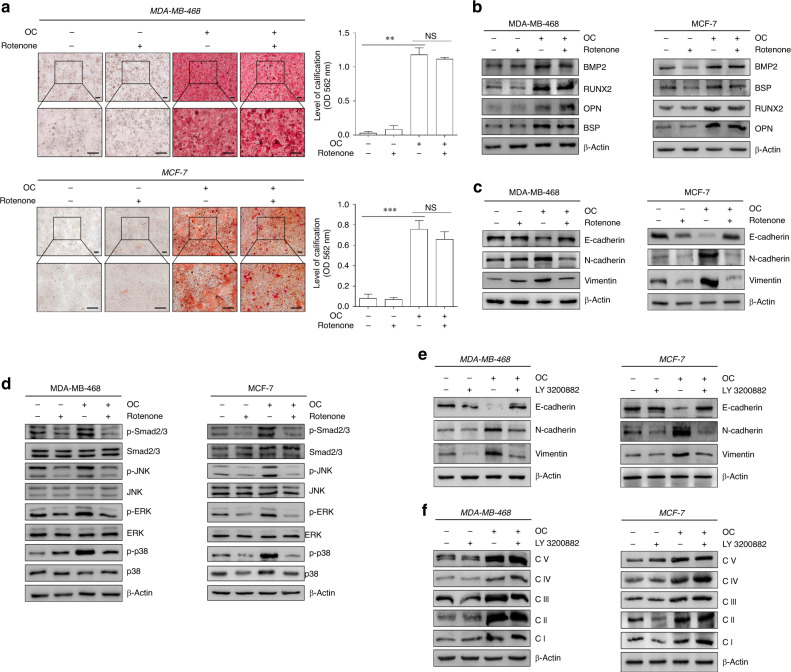
Alteration of OXPHOS activity is associated with aggressive cancer phenotypes in breast cancer cells during osteogenic differentiation. MCF-7 and MDA-MB-468 cells were treated with OC for 5 and 7 days, respectively; 2 µM rotenone (complex I inhibitor) was added during the last 24 h of culture. **a** Alizarin red S staining was performed in different treatment groups, and quantitative analysis of calcification was conducted using cetylpyridinium chloride. *P* value was calculated by one-way ANOVA with Bonferroni post-test. **P < 0.01, NS not significant. **b** Western blotting was performed to determine the expression of calcification-related markers in MDA-MB-468 and MCF-7 cells. **c** The corresponding expression of epithelial-to-mesenchymal transition (EMT)-related molecules was evaluated by immunoblotting in MDA-MB-468 and MCF-7 cells. **d** Cell extracts were subjected to western blotting with the antibodies against phospho-Smad2/3, phospho-JNK, phospho-ERK, phospho-P38, Smad2/3, JNK, ERK, P38 and β-actin. **e**, **f** MCF-7 and MDA-MB-468 cells were treated with OC for 5 and 7 days, respectively; 10 µM LY 3200882 (transforming growth factor (TGF)-β inhibitor, Selleck) was added during the last 24 h of culture. The corresponding expression of EMT-related molecules and OXPHOS complex subunits was evaluated by western blotting. Data are shown as mean ± SD, and they represent three independent experiments with similar results.

To investigate the importance of OXPHOS in pathological breast cancer calcification, we treated breast cancer cells with the OXPHOS inhibitor rotenone during osteogenic differentiation. Interestingly, rotenone treatment did not influence osteogenic differentiation in breast cancer cells in response to OC administration (Fig. 6a, b). However, OXPHOS inhibition abrogated the increased EMT process in OC-treated cells (Fig. 6c). Upon inhibition of OXPHOS, activation of TGF-β/Smad and MAPK signalling was comparable between OC-treated and control cells (Fig. 6d). TGF-β inhibition abolished the increased EMT process in OC-induced cells. Surprisingly, it did not affect the enhancement of the OXPHOS complex in response to OC (Fig. 6e, f). Thus, OXPHOS acts as a potential intermediate bridge between calcification and EMT. This indicates that calcification enhances the OXPHOS process and subsequently activates TGFβ-related signalling pathways, ultimately facilitating EMT.

## Discussion

Breast calcifications are small calcium deposits that develop within the breast tissue of women. These calcifications are common and are usually noncancerous (benign). In some instances, certain patterns of calcifications, such as tight clusters with irregular shapes and fine appearance, may indicate early breast cancer. Different calcification characteristics may occur in various types of breast cancer, indicating that compared with breast cancer cell MDA-MB-231 (triple-negative), MCF-7 (luminal type) and MDA-MB-468 (basal-like) cells might have more competency for pathological microcalcification. Wang et al. and Seo et al. reported that HER2/neu-overexpression breast carcinoma is also associated with mammographic calcification.^31,32^ In this study, we observed the association of mammographic calcification with the poor prognosis of patients with breast cancer by evaluating the clinical perspective of the relationship between mammographic calcification features and clinical–pathological factors in breast carcinomas. We also observed that the formation of microcalcifications in breast cancer cells is associated with their migration capabilities. The Kaplan–Meier analysis may not be fully consistent with the data in vitro. Confusion factors (e.g., sample size, follow-up time, evaluation criteria and analysis methods) may affect the results of the analysis. The patient followed up in the Kaplan–Meier analysis may be affected by genetics and external factors. The current research development trend is a combination of multidisciplinary and multicentric aspects, and it is optimal and convincing to evaluate and verify the prognostic outcome from multiple perspectives and dimensions. Mechanistically, this study proved that calcification enhances mitochondrial OXPHOS in breast cancer cells, thereby promoting TGF-β/Smad signalling and facilitating tumour metastasis.

Microcalcifications are usually not a result of cancer, but could indicate that a woman is at risk for developing breast cancer. Notably, breast calcifications show up on mammograms as bright white specks or dots on the soft-tissue background of the breasts, which makes mammography a common method for breast cancer screening. The main malignant component of mammary microcalcification is HA. Therefore, the osteogenic mixture used in this study contained HA calcium, which has emerged as a well-known in vitro model for the study of calcification. The formation of calcification and alterations in tumour metastatic capacity are inseparable from the comprehensive effects of the following three ingredients in the OC: β-glycerophosphate, which is the most important constituent for the development of HA crystals and cancer progression, and ascorbic acid and dexamethasone, which may play auxiliary and synergistic roles in the whole process to form HA crystals in a relatively short time.^33,34^ The deposited HA may be capable of stimulating the migration of the surrounding cells within the tumour microenvironment, further contributing to the progression of breast cancer.^35^ The data showed that breast cancer with osteogenic features is more likely to metastasise, which is consistent with the poor prognosis of breast cancer with clinical microcalcification. Rizwan et al. reported a correlation between increased calcium deposition and the ability of breast cancer cells to metastasise to distant organs.^36^ Another study demonstrated that the expression of some calcification-related genes was associated with bone metastasis.^17–19^ Bone is the most common site for breast cancer metastasis, observed in up to 70% of patients with metastatic disease.^37^ Current research on animal models of breast cancer calcification is relatively scarce.^38,39^ The results of our study highlight that calcification induces EMT in breast cancer cells, revealing a potential mechanism to explain the relationship between calcification and tumour metastasis.

Metabolism is also a hallmark of tumour transformation. It has been reported that calcium signalling regulates various key processes in tumorigenesis, such as proliferation, migration, invasion, cell death and angiogenesis.^40^ Breast cancer with calcification may be the result of tumour necrosis, or it may be a product secreted by the tumour. It may also involve calcium homoeostasis, which is associated with metabolic alteration.^6^ Alterations in the expression and/or activity of Ca^2+^-permeable ion channels are characteristic of certain breast cancer cells. Most studies have more focused on assessing the role of calcium signalling in key cancer progression, or identifying the alteration of specific calcium-permeable channels in breast cancer cells and the mechanisms involved. Our study highlights the emerging areas of metabolic changes during breast cancer calcification, especially the significantly enhanced mitochondrial OXPHOS.

Emerging evidence indicates that EMT is associated with a complex metabolic reprogramming in cancer cells.^41,42^ In some circumstances, alterations of metabolism could modulate EMT in cancer cells.^42^ Malignant tumour cells regulate their glucose metabolism to enhance aerobic glycolysis, thus maintaining their metastatic potential.^43^ Hexokinase 2 (HK2) is a key enzyme that catalyses the first committed step of glucose metabolism.^44^ In cancer cells, HK2 is often strongly expressed as a result of augmented glucose metabolism.^44,45^ Here, we observed that HK2 level was markedly increased in breast cancer cells upon osteogenic differentiation, indicating that calcification enhanced glucose metabolism in cultured breast cancer cells. HK2 expression is significantly upregulated in human brain metastatic derivatives of breast cancer cells.^46^ Notably, HK2 overexpression was significantly correlated with advanced-stage cancers, and served as an independent prognostic factor. Elevated HK2 level facilitates EMT through an enhanced glycolytic phenotype in tongue squamous cell carcinoma.^47^ HK2 knockdown significantly impeded the migration and invasion of ovarian cancer cells.^44^ The enhanced glucose metabolism might promote tumorigenesis by providing energy. Glycolysis and OXPHOS cooperate to maintain the cellular energetic balance in cancer cells.^48,49^ Lactate dehydrogenase A (LDHA), which has a higher affinity for pyruvate, is generally associated with high rates of aerobic glycolysis. PGC-1α participates in mitochondrial biogenesis and OXPHOS in cancer cells to promote metastasis.^50^ Our data showed a significantly increased expression of PGC-1α in breast cancer cells in response to osteogenic differentiation. The underlying molecular mechanism of OXPHOS in the metastasis of calcified breast cancer has attracted our attention. Surprisingly, the EMT phenotype was reversed, and the calcification-related indicators did not change significantly when OXPHOS activity was blocked. OXPHOS may be the intermediate bridge between calcification and EMT. By contrast, incubation with OC did not influence the level of LDHA in breast cancer cells. These results indicated that osteogenic differentiation mainly affects OXPHOS to meet the increased energy demand during osteogenic differentiation. This finding is in agreement with that of a previous study by Signorile et al., which suggested that OXPHOS and increased mitochondrial activity were essential for the metastatic phenotype.^51^ Numerous studies have investigated the effects of disorders arising owing to altered mitochondrial OXPHOS on EMT induction.^52,53^ Increased invasiveness of breast cancer cell lines is associated with impaired mitochondrial function.^49^ Jia et al. observed that compared with non-metastatic isogenic 67NR cells, highly metastatic mouse breast cancer 4T1 cells displayed enhanced OXPHOS activities.^53^ Mitochondria were initially considered as “calcium-sinks”, and mitochondrial calcium uptake plays a crucial role in the maintenance of cellular energy homoeostasis.^54^ Therefore, the modulation of metastasis of breast cancer cells could be partially attributed to intracellular calcium transport; further investigations in this regard are warranted.

Mitochondrial dysfunction also induces the phosphorylation of c-Jun/AP-1, which is recognised as an inducer of TGF-β. Here, we found that TGF-β expression was significantly upregulated in breast cancer during the calcification-induction process. It has been reported that the alteration of intracellular calcium concentration could regulate EMT induced by TGF-β1 through the Smad signalling pathway.^55^ We also observed the activation of the TGF-β/Smad pathway in OC-cultured cancer cells. In addition, the MAPK pathways were activated in the calcification process, which supports that the MAPKs regulate EMT in tumour metastatic processes.^56^ The non-Smad pathways can also be activated by the TGF-β receptors through phosphorylation or direct interaction. These pathways include various branches of MAPK, Rho-like GTPase signalling and PI3K/AKT pathways.^57^ ERK, JNK and Rho A regulate TGF-β-induced migration in MCF-7 cells, as well as in the SMAD4-deficient breast cancer cell line MDA-MB-468.^58^ The cooperation between SMAD and non-SMAD signalling pathways determines the outcome of the cellular response to TGF-β.^59^ Cancer therapies targeting metabolic pathways have been investigated for many years. It has been hypothesised that various metabolites provide fuel for subsequent mitochondrial metabolism, which is also called promoting mitochondrial metabolism, thereby providing more energy reserves for metastasis. Blockade of OXPHOS activity significantly inhibited the activation of TGFβ and MAPK signalling pathways, and further reversed the EMT phenotype of breast cancer cells upon osteogenic differentiation. These findings provide a novel direction/new perspective to explore new metabolic regulators for OXPHOS that might limit the metastasis of breast cancer.

## Supplementary information

Supplementary files

## Data Availability

All data generated or analysed during this study are included in this paper.
