# Supplementary figures and images for: OXPHOS-dependent metabolic reprogramming prompts metastatic potential of breast cancer cells under osteogenic differentiation
Source: Br J Cancer. 2020 Sep 16;123(11):1644–55. doi: 10.1038/s41416-020-01040-y (PMC7686370; doi:10.1038/s41416-020-01040-y)

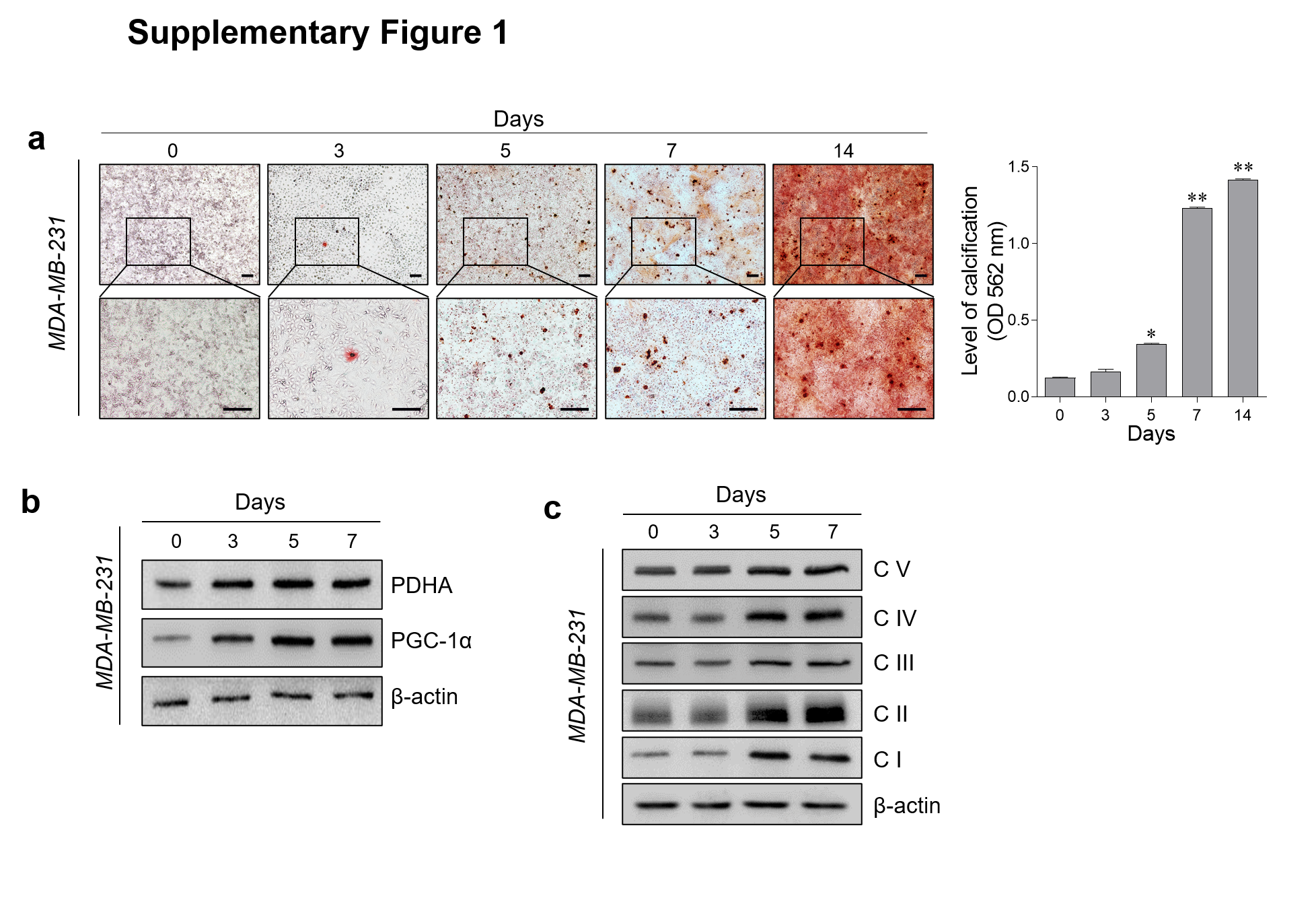

Supplement: Supplementary file 1 — Supplementary files [file 41416_2020_1040_MOESM1_ESM.zip › Supplementary files提交版/Supplementary files/Supplementary Fig. 1 Enhancement of mitochondrial metabolism in the calcification-mediated EMT model.tif]

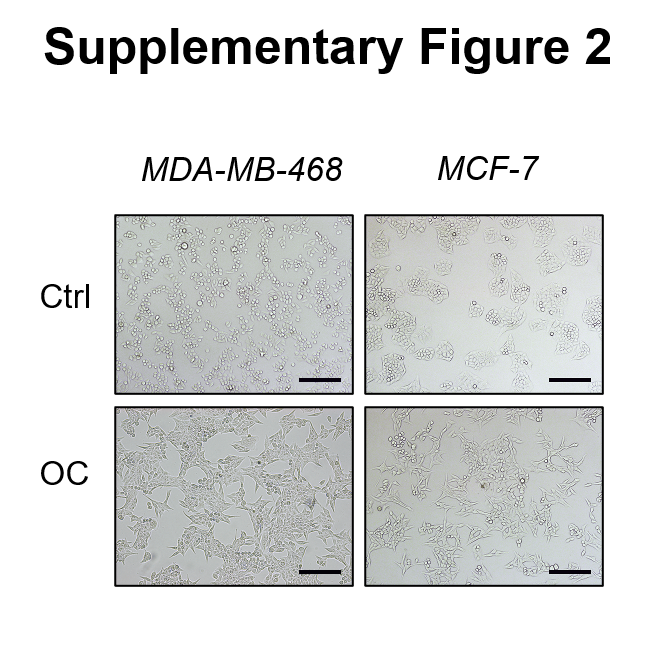

Supplement: Supplementary file 1 — Supplementary files [file 41416_2020_1040_MOESM1_ESM.zip › Supplementary files提交版/Supplementary files/Supplementary Fig. 2 Alteration of cell morphology in the calcification-mediated EMT model.tif]

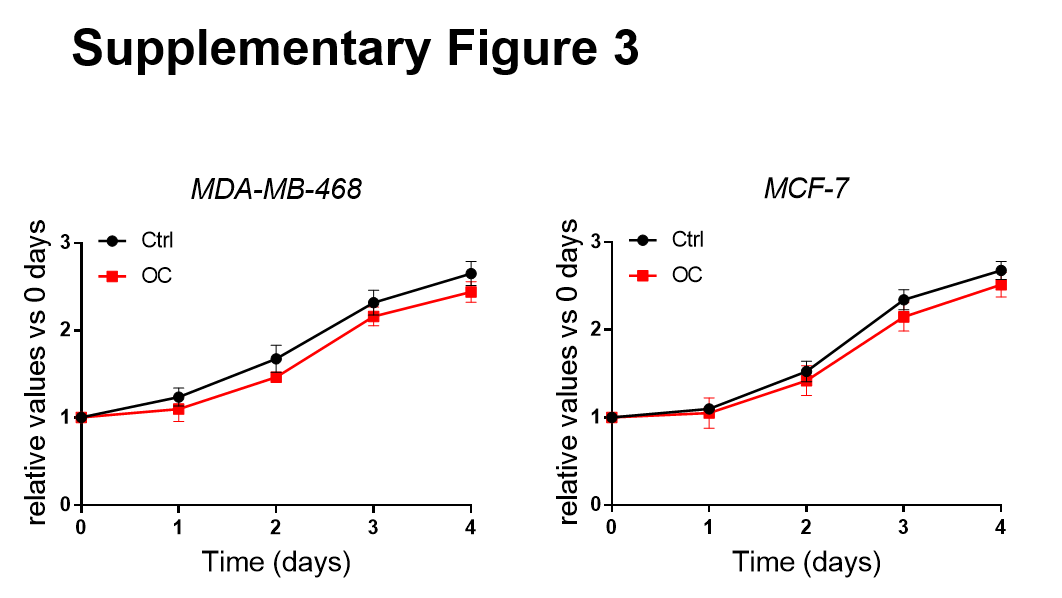

Supplement: Supplementary file 1 — Supplementary files [file 41416_2020_1040_MOESM1_ESM.zip › Supplementary files提交版/Supplementary files/Supplementary Fig. 3 Minor alteration in cell proliferation detected by CCK8 assay during calcification.tif]
