# Supplementary material for: OXPHOS-dependent metabolic reprogramming prompts metastatic potential of breast cancer cells under osteogenic differentiation
Source: Br J Cancer. 2020 Sep 16;123(11):1644–55. doi: 10.1038/s41416-020-01040-y (PMC7686370; doi:10.1038/s41416-020-01040-y)
Supplement: Supplementary file 1 — Supplementary files [file 41416_2020_1040_MOESM1_ESM.zip › Supplementary files提交版/Supplementary files/Supplementary figure legends.doc]

**Supplementary Materials**

**Supplementary Fig. 1 Enhancement of mitochondrial metabolism in the calcification model**

**(a)** Calcification of breast cancer cell line MDA-MB-231 was measured with alizarin red S staining at different time points after OC administration. Scale bars = 200 µm. **(b–c)** The expressions of mitochondrial relative genes (e.g., PDHA, PGC-1α) and OXPHOS complex subunits of MDA-MB-231 cells at the corresponding induction time were evaluated by western blotting. β-actin was used as an internal control.

**Supplementary Fig. 2 Alteration of cell morphology in the calcification-mediated EMT model**

MDA-MB-468 and MCF-7 cells were plated into 35-mm dishes at a density of 5 × 10^4^/mL. After incubation in the ordinary medium or OC medium for 3–4 days, cell morphology was observed under the microscope, and photographs of five random fields were captured. Scale bar = 200 µm

**Supplementary Fig. 3 Minor alterations in cell proliferation detected by CCK-8 assay during calcification**

Cell proliferation was detected by CCK-8 assay in MDA-MB-468 and MCF-7 cells. Cells were treated with or without OC for 4 days.

NS, not signiﬁcant, determined by unpaired Student’s *t*-test. Data are shown as mean ± SD, and they represent three independent experiments with similar results.

**Supplementary Table 1.** Clinical and pathologic characteristics of patients with breast cancer according to their calcification status (n = 404)

**Supplementary Table 2.** Univariate and multivariate analyses of factors associated with bone metastasis in patients with breast cancer

**Supplementary Table 3.** Primer sequences used for qRT-PCR
